# Biodegradation of cyanide by a new isolated strain under alkaline conditions and optimization by response surface methodology (RSM)

**DOI:** 10.1186/2052-336X-12-85

**Published:** 2014-05-12

**Authors:** Shabnam Mirizadeh, Soheila Yaghmaei, Zahra Ghobadi Nejad

**Affiliations:** 1 Department of Chemical and Petroleum Engineering, Biotechnology Research Center, Sharif University of Technology, Tehran, Iran

**Keywords:** Cyanide, Biodegradation, Response surface methodology, Alkaline conditions

## Abstract

**Background:**

Biodegradation of free cyanide from industrial wastewaters has been proven as a viable and robust method for treatment of wastewaters containing cyanide.

**Results:**

Cyanide degrading bacteria were isolated from a wastewater treatment plant for coke-oven-gas condensate by enrichment culture technique. Five strains were able to use cyanide as the sole nitrogen source under alkaline conditions and among them; one strain (C2) was selected for further studies on the basis of the higher efficiency of cyanide degradation. The bacterium was able to tolerate free cyanide at concentrations of up to 500 ppm which makes it a good potentially candidate for the biological treatment of cyanide contaminated residues. Cyanide degradation corresponded with growth and reached a maximum level 96% during the exponential phase. The highest growth rate (1.23 × 10^8^) was obtained on day 4 of the incubation time. Both glucose and fructose were suitable carbon sources for cyanotrophic growth. No growth was detected in media with cyanide as the sole carbon source. Four control factors including, pH, temperature, agitation speed and glucose concentration were optimized according to central composite design in response surface method. Cyanide degradation was optimum at 34.2°C, pH 10.3 and glucose concentration 0.44 (g/l).

**Conclusions:**

Bacterial species degrade cyanide into less toxic products as they are able to use the cyanide as a nitrogen source, forming ammonia and carbon dioxide as end products. Alkaliphilic bacterial strains screened in this study evidentially showed the potential to possess degradative activities that can be harnessed to remediate cyanide wastes.

## Background

Cyanide is a group of compounds that contains the C ≡ N group. It is widely distributed in the environment. In water and soil systems, cyanide occurs in various physical forms including many different kinds of species dissolved in water, many different solid species and several gaseous species. Cyanide is a very toxic compound that is discharged into the environment through the effluents of industrial activities such as metal plating, electronics, photography, coal coking, plastics, production of organic chemicals and mining [1-3]. The toxicity of cyanide is quite high due to its ability to poison the respiratory system by inhibiting the final transport of electrons from cytochrome C oxidase to oxygen, preventing production of ATP. Exposure to small amounts of cyanide can be deadly irrespective of the route of exposure [4-7]. Because of the high degree of toxicity in certain forms of cyanide, primarily hydrogen cyanide (HCN), acceptable levels of cyanide compounds in water and soil are generally very low. For example, the U.S. drinking water maximum contaminant level for free cyanide (HCN and CN^-^) is 0.2 mg/l, while the U.S. ambient water quality criterion for acute exposures in freshwater systems is 22 μg/l . As this thousand-fold difference indicates, some aquatic organisms are significantly more sensitive to cyanide than are humans [2,7,8]. Cyanide waste is becoming an increasingly prevalent problem in today’s society. To protect the environment and water bodies, wastewater containing cyanide must be treated before discharging into the environment. There are several conventional methods used in treating effluents containing cyanide before discharging it into the environment. The most common ones are the alkaline chlorination, sulfur oxide/air process and hydrogen peroxide process [6,9]. However, these methods are expensive and hazardous chemicals are used as the reagents (chlorine and sodium hypochlorite) and Some of them create additional toxic and biological persistent chemicals. Despite cyanide‘s toxicity to living organisms, biological treatments are feasible alternatives to chemical methods without creating or adding new toxic and biologically persistent chemicals [1,10-12]. The biological treatment relies upon on the acclimation and enhancement of indigenous microorganisms such as bacteria, but most of the time the environmental conditions, mainly the chemical composition, must be previously modified. Several studies have been established for the use of bacterial strains such as *Pseudomanas, Acinetobacter, Burkhoderia cepacia* and *Alcaligenes* spp., *Bacillus nealsonii*[13]*Serretia marcescens*[14]*Streptomyces phaeoviridae* as the useful *Actinomycetes*[15] and only few algae like *Arthrospira maxima*, *Scenedesmus obliquus* and *Chlorella* spp. [10,16]. Recently a basidiomycetous yeast *Cryptococcus cyanovorans* sp. nov., has been isolated from cyanide contaminated soil [17]. A new bacterial strain, *Rhodococcus* UKMP-5 M isolated from petroleum-contaminated soils demonstrated promising potential to biodegrade cyanide to non-toxic end-products [18]. Some microorganisms have been described to be able to degrade cyanide at a neutral or acidic conditions, but under this condition a high concentration of cyanide evaporates as hydrocyanic acid (HCN), a weak acid with a pKa value of 9.2 [16]. Thus, it is very important to isolate cyanotrophic microorganisms that function at alkaline pH. Cyanide biodegradation at alkaline pHs is less mentioned in the references. For example the bacterial strain *Pseudomonas pseudoalcaligenes*, which uses cyanide as the sole nitrogen source is mentioned [19]. The bacterium *Burkholderia cepacia* is able to remove cyanide in a pH range from 8 to 10, with a maximum cyanide removal (1.85 mg CN.h-1) at pH 10 [20]*.* A fungus *F.solani*[21] under alkaline conditions (pH 9.2 –10.7) demonstrated that the cyanide was degraded via a cyanide hydratase and amidase pathway. Cyanide degradation by the strain CECT5344 in reactors operating at a constant pH of 9.5 may thus provide an effective alternative to existing physico-chemical treatments for the detoxification of wastewater containing cyanide or cyano-metal complexes with the need for no chemical pre-treatment. The aim of this study was isolate and identify cyanide-degrading bacteria under alkaline conditions from a wastewater treatment plant for coke-oven-gas condensate in the Esfahan Steel Company and optimize operational conditions by RSM. Such microorganisms are the ones suitable for practical applications of cyanide biodegradation. Therefore, continuous search for cyanotrophic microorganisms capable of degrading cyanide at alkaline conditions is the principal element of the effort being made to develop efficient biotechnological methods of cyanide removal.

## Methods

### Isolation of cyanide degrading microbes

Cyanide-degrading microorganisms were isolated from a wastewater treatment plant for coke-oven-gas condensate in the Esfahan Steel Company and purified by repeatedly transferring the cells to enrichment medium. Nutrient broth was used for enrichment of microorganisms. The sample was cultivated in a 500 ml Erlenmeyer flask containing 100 ml nutrient broth, with Cyanide Concentration Changes from 30 to 100 mg/l. The enrichment of cyanide degrading microorganisms was conducted by sub culturing every 3 days for 2 weeks with 10% (v/v) inocula in a rotary shaker at 150 rpm and 30°C. The pH is intentionally kept highly alkaline on or above 9.5 to minimize volatilization of cyanide as HCN. In order to screen cyanide degrading bacterium, 10 ml of culture was transferred into 500 ml Erlenmeyer flask containing 100 ml of buffer medium (BM) and 100 mg/l CN added and incubated at 30°C, 150 rpm. Process was repeated three times by reinoculation in fresh medium with 10% (v/v) of the previously grown culture. After 4 days, cyanide-degrading bacteria were isolated done by streaking on nutrient agar medium. Colonies differing mainly in the morphology were selected and pure cultures were obtained by continuous sub-culturing. Isolated bacteria were tested for their Gram reactions, and other physiological and biochemical tests, such as catalase and oxidase, were performed [22].

### Media condition

Buffer medium (BM) was used as media in this study. 1 liter of BM contained K2HPO4 4.35 g, NaOH 4 g and 10 ml of trace salts solution (FeSO4•7H2O 300 mg, MgSO4•7H2O 180 mg, CoCl2 130 mg, CaCl2 40 mg, MnCl2•4H2O 40 mg and MoO3 20 mg in 1 liter deionized water) and 0.1% yeast extract. Before sterilization, the pH of the medium was adjusted to 9.5-10. The medium was autoclaved for 20 min at 15 psi and 121°C. Potassium cyanide from a filter-sterilized (0.2-mm-pore-size filter) solution was added to the medium as a nitrogen source and filter sterilized glucose (1 g/l) was routinely used as the carbon source after autoclaving.

### Cyanide degrading experiment

Distinct morphological colonies of bacterial strains were inoculated in nutrient broth for 24 hrs. For the purpose of strains comparison study, cyanide removal was determined after 72 hours of incubation with initial cyanide concentration of 200 mg/l in the BM. A single strain was selected on the basis of the greater efficiency of cyanide degradation. The selected bacterium was inoculated in BM containing KCN at 100, 200, 300, 350, 400, 450 and 500 mg/L in 500 ml Erlenmeyer flask and incubated at 30°C on a rotary shaker (150 rpm) for 2 weeks. Samples were taken at regular intervals and tested for cyanide reduction. Non-inoculated medium served as control. All experiments were carried out in triplicates with control.

### Bacterial growth analysis

The growth of isolated bacterium was studied by colony count technique. The number of viable colonies was determined daily by pour plate technique on nutrient agar for 7 days. 1 ml of isolated bacterium in BM containing 200 mg/L CN- was obtained from the flask and a ten-fold dilution was performed with sterile 0.9% NaCl solution. After that 1 ml of each dilution was pipetted into a sterile plate, and then melted agar was poured in and mixed with the sample. The plates were incubated at 30°C for 24 hours (each dilution plated in triplicate). The plates containing 30-300 colonies were counted and used for calculation of viable cell concentration as colony forming units/ml (CFU/ml) [23].

### Analytical methods

Residual cyanide was analyzed by DR 5000 Spectrophotometer UV-VIS and cyanide test kit (24302-00) according to the Method 8027 (Pyridine-Pyrazalone Method (0.002 to 0.240 mg/L CN^–^)) provided by the HACH company. The Pyridine-Pyrazalone method used for measuring cyanide gives an intense blue color with free cyanide. Test results are measured at 612 nm [24]. Ammonia (NH3) (Nesslerization spectrophotometric method) and Nitrate (NO^3-^) (spectrophotometric method, for use at 220 nm and 275 nm) were determined according to APHA standard methods [23]. The concentration of ammonia and nitrate were measured for the identification of final products.

### Optimization studies

Glucose, fructose, acetate sodium, sucrose were used to determine their effect on cyanide utilization by adding 10 ml of bacterial suspension to BM (100 ml) supplemented with each of the carbon sources and 200 mg/l CN. In similar experiments to determine the effects of nitrogen sources, the BM was separately supplemented with each of the following nitrogen source: ammonium sulfate, ammonium nitrate and urea to a concentration of 1 g/l and glucose as a carbon source inoculated with the bacterial suspension. The experimental design and statistical analysis were performed according to the RSM (Response Surface Methodology) using Design-Expert software (Trial Version 7.1.5, Stat-Ease, Minneapolis, 2008) for Optimization of growth parameters. Central composite experimental design (CCD), with quadratic model was employed to study the combined effect of four independent variables namely temperature (25 - 45°C), pH of medium (8 - 13), agitation rate (100-200 rpm) and carbon source concentration (1-10 g/l). A total of 30 runs are used to optimize the medium. Upon completion of experiments, residual cyanide concentration was taken as a depended variable or response Y. The experiments were conducted for 3 days.

## Results and discussion

### Isolation of microorganisms growing in the presence of cyanide

From a chemical point of view, the biological treatment of industrial effluents contaminated with cyanide requires an alkaline pH in order to avoid the volatile HCN (pKa = 9,2) formation [25]. Thus, the first step in the biological treatment process is the selection of bacteria able to tolerate and degrade cyanide in the millimolar range at alkaline pH. Five bacteria (named as C1-C5) were successfully and repeatedly isolated from coke oven wastewater by their ability to grow in media that had been supplemented with cyanide. Pure colonies were obtained and then each one was cultured for cyanide degradation. The microorganisms isolated were assayed in batch culture for their ability to biodegrade cyanide under alkaline conditions. At initial KCN concentration of 100 mg/l, the cyanide removal was about 76%, for all strains. The strain C2 (a gram- negative, aerobic rod) was selected on the basis of the greater efficiency of cyanide degradation (86%) after 3 days incubation, for further studies (Table 1). The incubation conditions were pH 10, 30°C, and the initial cyanide concentration was 200 mg/l. The growth of bacteria was observed through increasing the turbidity of culture medium without yeast extract (cyanide analysis is not assayed). But the strain C2 was able to degraded cyanide concentration of about 57%, therefore bacterial strains are capable use cyanide as the sole source of nitrogen. Addition of a small amount of yeast extract led only to microorganism growth. Along with the selected individual strains, a mixed bacterial consortium prepared using the above strains were also used for degradation studies. The mixed bacterial consortium showed less growth and degradation than the strain C2. The mixed culture was able to degraded cyanide concentrations of about 67%, while the strain C2 degraded concentrations up to 86%. The pure cultures were found to be more efficient than the mixed cultures at reducing the free cyanide. This possibly happens due to inter- and/or intra species interaction amongst the bacteria or various groups of microorganism which can increase accumulation of inhibitory by-product of cyanide breakdown. Favorably, bacterial strains in this study are capable of degrading and tolerating higher concentrations at alkaline condition well above pKa value of cyanide.

**Table 1:** Cyanide removal efficiency by isolated bacteria

**Strains**	**Cyanide removal efficiency %**
C1	45
C2	86
C3	62
C4	73
C5	67

### Cyanide degrading experiment

**Figure 1:**
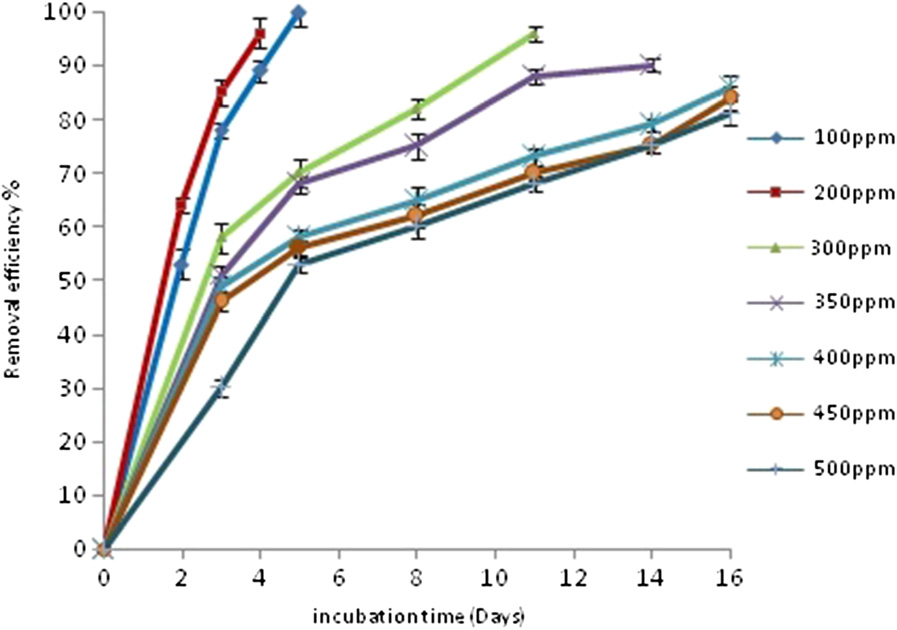
**The effect of initial cyanide concentration (CN**_**free**_**) on the degradation of cyanide by strain C2, The experiments were repeated three times.** (Temperature, 30°C; pH 10; agitation rate, 150 rev/min).

To determine the effects of the initial amount of cyanide concentrations, 100 to 500 mg cyanide/L was added to the solution. As the initial cyanide concentrations increased, percentage of cyanide degradation decreased (Figure 1). The optimum initial concentration was deemed as 200 mg cyanide/L. Cyanide was completely depleted after 4 days. However at high concentration (500 mg/L), only about 45% was degraded within 4 days. It might be because of the fact that microbial degradation starts slowly and requires an acclimation period before rapid degradation occurs in high concentration. In non-inoculated controls at different initial concentrations, the levels of cyanide did not decrease during the incubation period. There are studies reporting comparable or higher biodegradable cyanide concentrations [12,26-29]. However majority of these are taking place in acidic, natural or slightly alkaline conditions, below dissociation constant. Strains growth has been evaluated on cyanide as the only source of both carbon and nitrogen and was no significant cell count observed.

### Cyanide-degrading bacterial growth

**Figure 2:**
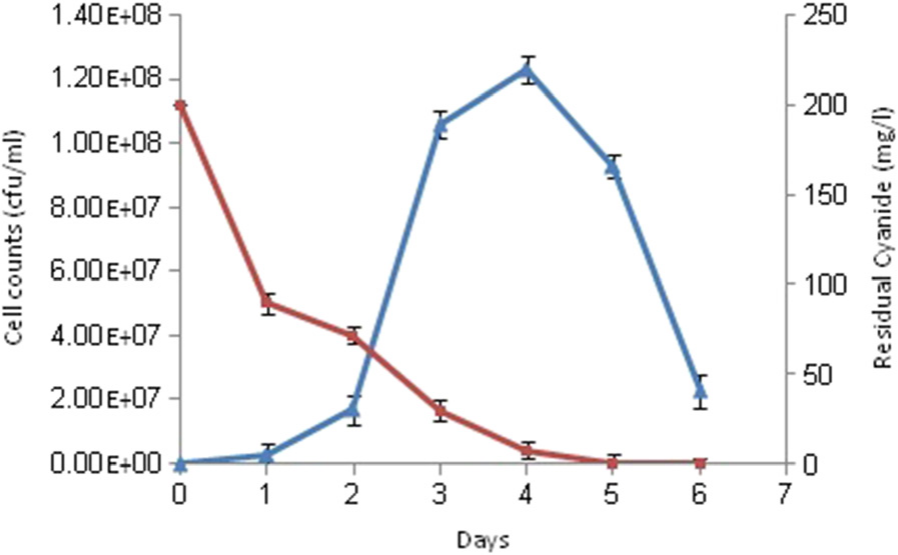
**Bacterial growth, and cyanide degradation by strain C2.** Bacterial growth with cyanide (▲), cyanide concentration (CN_free_) (■) The experiments were repeated three times.

The growth of strain C2 is shown in Figure 2. Cyanide utilization occurred mainly during the exponential phase of growth. The highest growth rate (1.23 × 10^8^) was obtained on day 4 of the incubation time. In all the media except the control, residual cyanide decreased significantly to lower levels during the incubation period. The control which was kept without inoculum was found to lose some amount of cyanide but it was relatively not significant. As can be seen from Figure 2, bacterial growth and cyanide removal were correlated throughout. These results clearly show that cyanide can be fully degraded by this strain.

According to the current knowledge, all of the microorganisms able to assimilate cyanide can use it only as a nitrogen source, but not as the sole carbon source. In the cyanide molecule, the oxidation state of C (+2, like that in CO) and N (-3, like that in NH4 +) make this compound a bad C source but a good N source for bacterial growth. Some microorganisms are able to grow in medium containing only cyanide compounds as nutrients (i.e. carbon and nitrogen). But other microorganism such as *Pseudomonas fluorescens* P70, *Bhurkholderia cepacia* strain C3 [20], could not grow in medium containing only cyanide as nutrient. In these cases there is a need to supply an external carbon source generally provided as glucose. [13-15] Among the carbon sources, fructose and glucose readily supported the utilization of cyanide, the highest cyanide utilizing activity occurring in the presence of glucose. With acetate sodium and sucrose poor cyanide utilization was observed. The medium supplemented with nitrogen sources, cyanide biodegradation inhibited. The incubation conditions were pH 10, 30°C, and the initial cyanide concentration was 200 mg/l (Table 2).

**Table 2:** Effects of different carbon and nitrogen sources on cyanide removal

**Carbon source**	**Removal efficiency of cyanide %**	**Nitrogen source**	**Removal efficiency of cyanide %**
Fructose	82	Ammonium sulfate	18
Sodium acetate	72	Ammonium nitrate	14
Sucrose	57	Urea	17
Glucose	85		

Microorganisms are able to convert cyanide into other less toxic products like ammonia, formic acid and formamide depending upon the enzyme system they possess As shown in Table 3, strain C2 is able to degrade cyanide to ammonia and nitrate. The background of biological removal of cyanide compounds showed a production of ammonia during the degradation of cyanide by the microorganisms. Therefore, the assessment of ammonia concentration in the sample could be strong parameters to prove the biodegradation of cyanide compounds. The ammonia revealed the increasing concentration when the cyanide removal efficiency increased. Cyanide can be degraded to ammonia and converted finally to nitrate as a final by-product [28]. Therefore strain C2 could be used for the treatment of many industrial alkaline effluents and bioremediation of cyanide-containing waste. However, the others by-product such as methane, carbon dioxide, or nitrite can be occurred.

**Table 3:** Cyanide degradation at 200 mg/l of cyanide concentration

**Cell counts CFU/ml**	**pH**	**NO**_**3**_^**-**^**(mg/l)**	**NH**_**3**_**(mg/l)**	**Residual cyanide (CN**_**free**_**) mg/l**	**Removal efficiency %**	**Time (Days)**
1.3 × 10^6^	10.2	0.0	0.0	200	0	0
1.7 × 10^7^	9.8	3.6	1.7	72	64	2
1.06 × 10^8^	10	4.1	2.3	30	85	3
1.23 × 10^8^	10.1	6.3	-	8	96	4

### Response surface analysis for the optimization

The Response Surface Methods (RSM) was used in evaluation of optimization of biodegradation cyanide. This method is based on analysis of response as affected by some factors and its objective is to determine the optimum condition of the response. The important factors which affect cyanide biodegradation were the temperature, initial pH, agitation rate and carbon source concentration (glucose). The design and observed values of residual cyanide concentration are presented in Table 4. Based on these results the model can be utilized to generate response surfaces and contour curves that indicated the effects of the factors on the cyanide degradation. The respond surfaces were fitted with Eq. (1):

(1) $$\begin{array}{l} Y=30.83+5.88A+33.71B‒1.88C\\ \;+5.69D‒0.56\mathrm{AB}‒0.19\mathrm{AC}‒0.19\mathrm{AD}\\ \;+0.44\mathrm{BC}‒0.56\mathrm{BD}+0.81\mathrm{CD}+16.74\;A^{2}\\ \;+26.36B^{2}+2.36C^{2}+5.11D^{2} \end{array}$$

**Table 4:** Experimental and predicted contents by RSM for cyanide concentrations

**Run no.**	**A:Temperature (°C)**	**B (pH)**	**C: Agitation rate (rpm)**	**D: Glucose concentration (g/l)**	**Removal efficiency %**	
					**Experimental**	**Predicted**
1	40	9.25	175	0.78	76	73
2	30	9.25	175	0.78	80	79
3	30	11.75	125	0.33	47.5	46.2
4	35	10.50	150	0.55	76.25	84.58
5	35	10.50	150	1.00	68.5	68.4
6	30	9.25	125	0.33	83	83.75
7	30	11.75	125	0.78	34.5	38.66
8	35	10.50	200	0.55	84	84.58
9	35	10.50	150	0.55	84	84.58
10	35	10.50	150	0.55	84	84.58
11	35	10.50	150	0.55	85	84.58
12	40	9.25	125	0.78	72.5	71.4
13	40	11.75	175	0.78	33	35.08
14	40	11.75	175	0.33	36	41.35
15	25	10.50	150	0.55	62.5	62.57
16	35	10.50	150	0.10	85.5	85.68
17	30	11.75	175	0.33	37.5	44.35
18	40	9.25	175	0.33	80.5	80.25
19	40	9.25	125	0.33	78	76.93
20	35	10.50	100	0.33	82	80.85
21	45	10.50	150	0.55	43	46.4
22	35	13	150	0.55	6.5	-6.6
23	40	11.75	125	0.78	33	34.88
24	35	8	150	0.55	59	55.7
25	40	11.75	125	0.33	39.5	36
26	35	10.50	150	0.55	84	84.6
27	30	11.75	175	0.78	35.5	40
28	30	9.25	175	0.33	87	87.8
29	30	9.25	125	0.78	79	77.85
30	35	10.50	150	0.55	85	84.6

Y is the predicted response (cyanide concentration), A (temperature), B (pH), C (agitation rate) and D (glucose concentration) are the independent variables.

**Figure 3:**
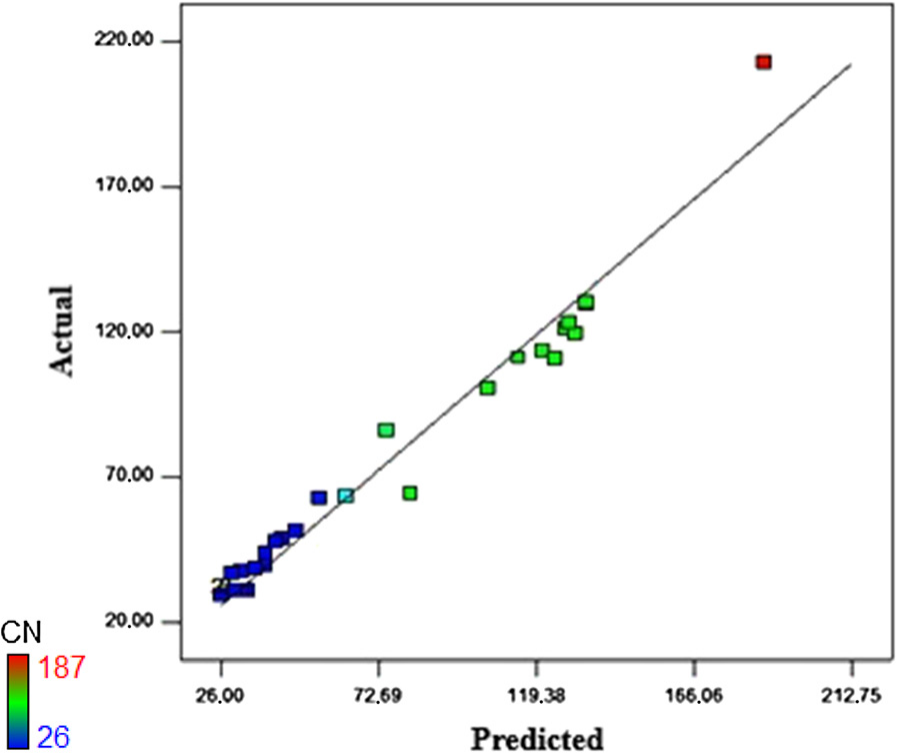
Parity plot showing the distribution of experimental vs. predicted values of cyanide content.

The predicted and experimental values of cyanide concentration are given in Table 5. Coefficient (R^2^) was calculated to be 0.9690, which can explain up to 96.90% variability of the response. R^2^ gives a measure of how much variability in the observed response value can be explained by the experimental factors and their interactions. The value of the adjusted determination coefficient (Adj R^2^ = 0.9401) is also high to advocate for a high significance of the model. The Pred R^2^ of 0.8220 is in reasonable agreement with the Adj R^2^. Adeq precision measures the signal to noise ratio. A ratio greater than 4 is desirable. In this work the ratio is 23.06, which indicates an adequate signal. The significance of each coefficient was determined by P-values which are listed in Table 5. The analysis of variance (ANOVA) of the quadratic model demonstrates that the model was highly significant. The probability of p-value for models of less than 0.05 indicates that significant models, less than 0.0001 indicates highly significant models and also greater than 0.1000 indicates the models were not significant. The interaction variable coefficients were found to be not significant in determining the response. A, B, D, A^2^, B^2^ and C^2^ are important model terms. In addition, the linear effect of B (pH) is the most important factor, while C (agitation rate) did not have a significant effect on the responses. As a result, the model, Eq. (1), might be modified into Eq. (2). Figure 3 shows a satisfactory correlation between predicted and experimental values.

(2) $$\begin{array}{l} Y=33.14+6.825A+37.08B+6.58D\\ \;+16.61A^{2}+26.61B^{2}+4.48D^{2} \end{array}$$

**Table 5:** Analysis of variance (ANOVA) for response surface quadratic model

**Source of variation**	**Mean square**	**F-value**	**p-value**
Model	4413.41	33.53	<0.0001
A-temperature	828.38	6.29	0.0241
B-pH	35960.04	273.17	<0.0001
C-agitation rate	84.38	0.64	0.4359
D-glucose conc.	852.04	6.47	0.0225
AB	5.06	0.038	0.84359
AC	0.56	0.0042	0.9487
AD	0.56	0.0042	0.9487
BC	3.06	0.023	0.8808
BD	5.06	0.038	0.8472
CD	10.56	0.080	0.7808
A^2^	7685.86	58.39	<0.0001
B^2^	19065.36	144.83	<0.0001
C^2^	153.30	1.17	0.2975
D^2^	717.50	5.45	0.0339

#### Interactions among the factors

**Figure 4:**
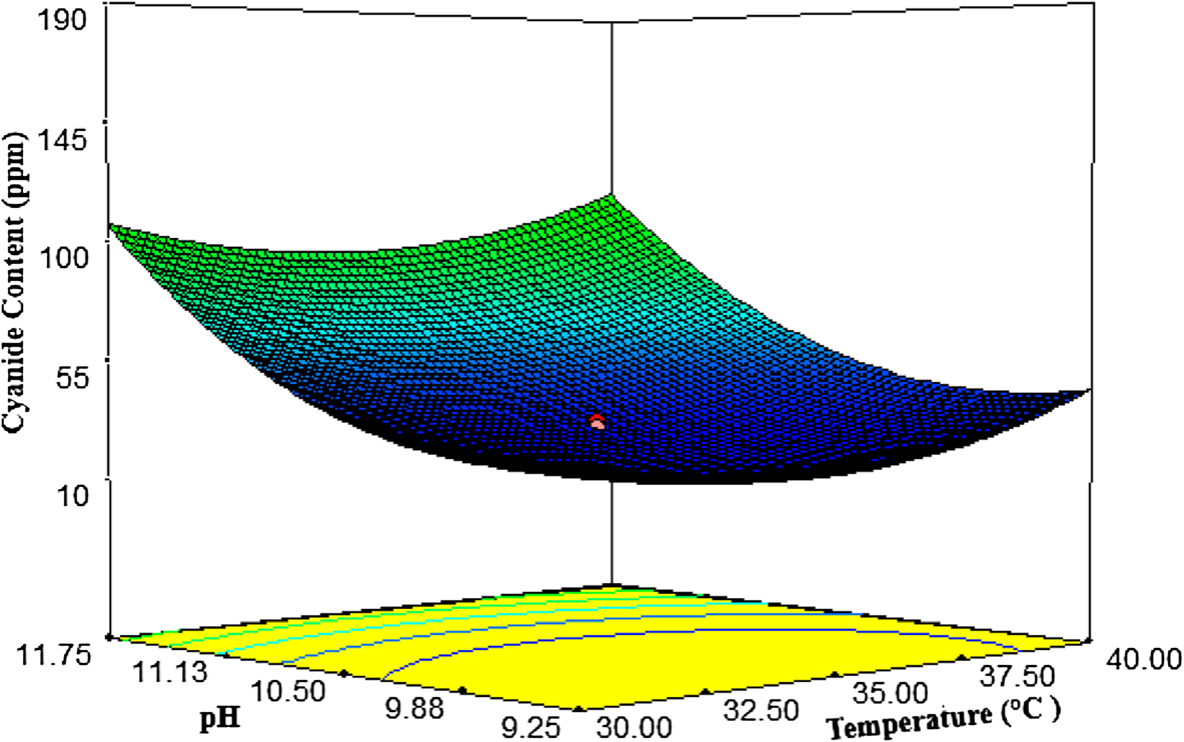
**Response surface plot of temperature vs. pH on cyanide content (CN**_**free**_**).**

**Figure 5:**
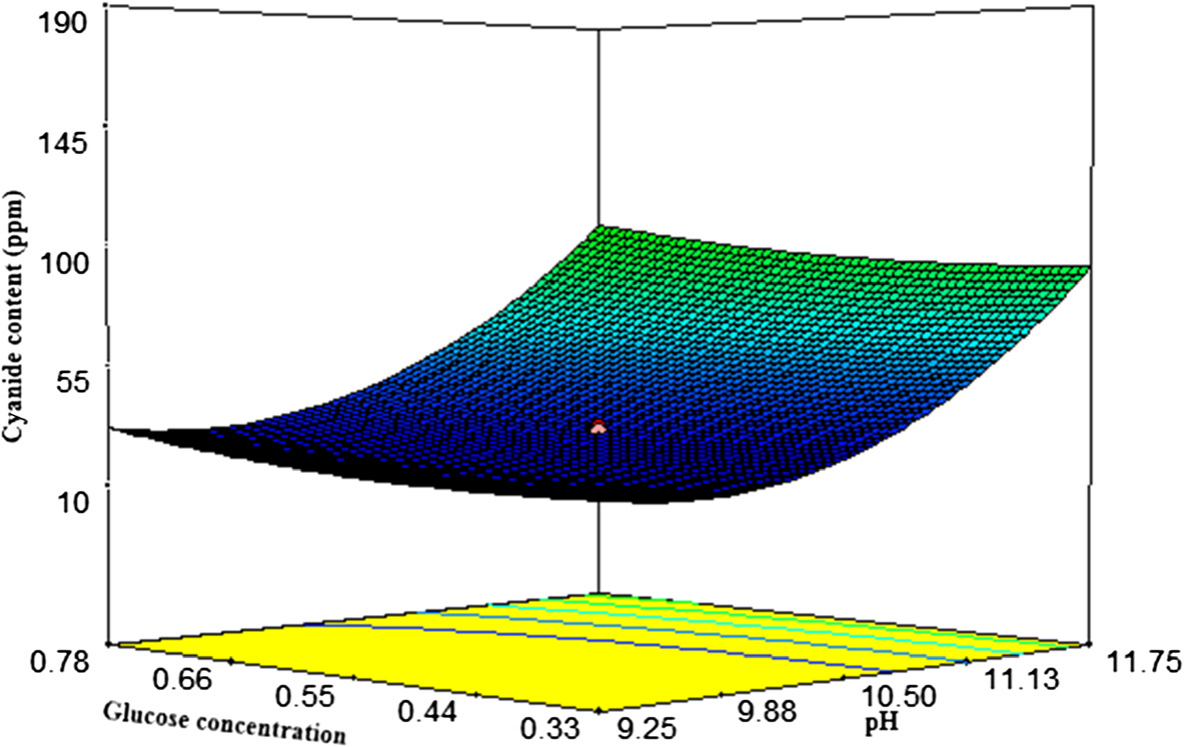
**Response surface plot of pH vs. glucose concentration on cyanide content (CN**_**free**_**).**

**Figure 6:**
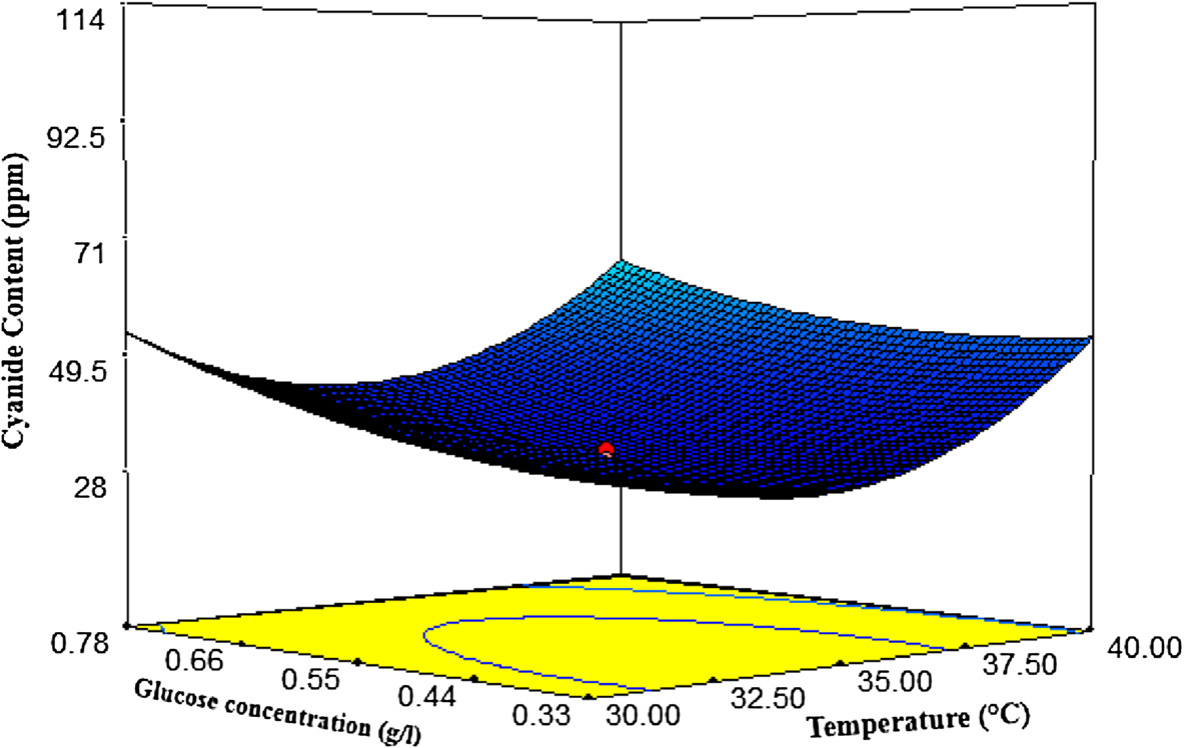
**Response surface plot of temperature vs. glucose concentration on cyanide content (CN**_**free**_**).**

Response surface plots as a function of two factors at a time, maintaining all other factors at fixed levels are more helpful in understanding both the main and the interaction effects of them. The response surface curves for cyanide content are shown in Figures 4, 5 and 6.These figures explain the effect of three independent variables; temperature, glucose concentration and pH on the cyanide concentration. Figure 4, shows the effects of pH and temperature on cyanide content. The cyanide content decreased with increasing temperature and pH in the range of 30 to 36°C and 9-10.5 respectively, however, the degradation rate was reduced for a further increase in temperature and pH value. The effects of glucose concentration and pH on cyanide content are shown in Figure 5. At higher pH range, glucose concentration did not have a significant effect on the responses. The interaction effects of glucose concentration and temperature on cyanide content (Figure 6) imply that the biodegradation cyanide should be carried out at a temperature in the range of 32 to 35°C for a glucose concentration of 0.44 to 0.55 (g/l) to achieve a minimum content of cyanide. The optimum conditions were at 34.23°C, pH 10.30 and glucose concentration 0.44 (g/l) which could provide 24 ppm for predicted cyanide content and 26 ppm for experimental content. The results showed that the model, Eq. (2), could be useful.

## Conclusions

It is well known that biological treatment of cyanide by utilizing indigenous microorganisms can be less expensive and more environmentally friendly for cyanide removal from aqueous solution compared with conventional techniques for instance chemical methods. The present study primarily focused on the isolation and purification of the microorganisms which utilize cyanide as the sole source of nitrogen in alkaline conditions. The strain C2 was selected on the basis of the higher efficiency of cyanide degradation (86%) after 3 days incubation. The strain was able to grow in alkaline media, up to an initial pH of 10.5, and tolerated free cyanide in concentrations of up to 500 ppm which makes it a good candidate for the biological treatment of cyanide contaminated residues. However, further studies such as its interactions with the environment, toxicological aspects, degradation enzymes, biochemical and genetic aspects are still needed before the application in actual field-scale bioremediation.

## Competing interests

The authors declare that they have no competing interests.

## Authors’ contributions

SHM, carried out this study as part of her M.Sc. project, collected the data, performed the analysis, and wrote the manuscript. SHM, ZG, SY, developed the methodology. ZG supervised the microbial part of this research, and revised the manuscript. SY, planed the original research of the project, supervised the whole project and read the manuscript. All authors read and approved the final manuscript.

## References

[B1] Dash RR Gaur A Balomajumder C Cyanide in industrial wastewaters and its removal: a review on biotreatment J Hazard Mater 2009 163 1 1 11 10.1016/j.jhazmat.2008.06.051 18657360

[B2] Dzombak DA Ghosh RS Wong-Chong GM Cyanide in Water and Soil: Chemistry, Risk, and Management 2005 London: Taylor & Francis Group

[B3] Baxter J Cummings SP The current and future applications of microorganism in the bioremediation of cyanide contamination Antonie Van Leeuwenhoek 2006 90 1 1 17 10.1007/s10482-006-9057-y 16683094

[B4] Westley J Vannesland B Conn EE Knowles CJ Wissing F Cyanide in Biology 1981 London: Academic Press Inc

[B5] Hagelstein K The Ecotoxicological Properties of Cyanide, in Short Course Notes on Management of Cyanide in Mining 1997 Perth: Australian Centre for Minesite Rehabilitation Research

[B6] Akcil A Mudder T Microbial destruction of cyanide wastes in gold mining: process review Biotechnol Lett 2003 25 6 445 450 10.1023/A:1022608213814 12882268

[B7] Taylor J Roney N Harper C ATSDR (Agency for Toxic Substances and Disease Registry) Toxicological Profile for Cyanide 2006 Atlanta, GA: U.S. Department of Health and Human Services, Public Health Service Published in Fed.Reg.

[B8] WHO Guidelines for Drinking-Water Quality 1984 Geneva: World Health Organization

[B9] Latkowska B Figa J Cyanide removal from industrial wastewaters J Environ Stud 2007 16 148 152

[B10] Gurbuz F Ciftci H Akcil A Karahan AG Microbial detoxification of cyanide solutions: a new biotechnological approach using algae Hydrometallurgy 2004 72 1–2 167 176

[B11] Ebbs S Biological degradation of cyanide compounds Curr Opin Biotechnol 2004 15 3 231 236 10.1016/j.copbio.2004.03.006 15193331

[B12] Akcil A Destruction of cyanide in gold mill effluents: biological versus chemical treatments Biotechnol Adv 2003 21 6 501 511 14499151 10.1016/s0734-9750(03)00099-5

[B13] Mohanraj Perumal JPJ Kamaraj M Isolation and characterization of potential cyanide degrading bacillus nealsonii from different industrial effluents Int J ChemTech Res 2013 5 5 2357 2364

[B14] Virender Kumar VK Tek Chand Bhalla In vitro cyanide degradation by Serretia marcescens RL2b Int J Environ Sci 2013 3 1969 1979

[B15] Shete HG Kapdnis BP Cyanide hydratase production using acclimatized strain of streptomyces phaeovride and its characterization Int J Bioassays 2013 8 1098 1103

[B16] Gurbuz F Ciftci H Akcil A Biodegradation of cyanide containing effluents by Scenedesmus obliquus J Hazard Mater 2009 162 1 74 79 10.1016/j.jhazmat.2008.05.008 18554792

[B17] Motaung TE Albertyn J Kock JLF Pohl CH Cryptococcus cyanovorans sp. nov., a basidiomycetous yeast isolated from cyanide-contaminated soil Int J Syst Evol Microbiol 2011 62 1208 1214 21828018 10.1099/ijs.0.034181-0

[B18] Nallapan Maniyam M Sjahrir F Ibrahim A Cass AG Biodegradation of cyanide by Rhodococcus UKMP-5 M Biologia 2013 68 2 177 185 10.2478/s11756-013-0158-6

[B19] Luque-Almagro VM Huertas MJ Martínez-Luque M Moreno-Vivián C Roldán MD García-Gil LJ Castillo F Blasco R Bacterial degradation of cyanide and its metal complexes under alkaline conditions Appl Environ Microbiol 2005 71 940 947 10.1128/AEM.71.2.940-947.2005 15691951 PMC546731

[B20] Adjei MD Ohta Y Isolation and characterization of a cyanide utilizing Burkholderia cepacia strain J Microbiol Biotechnol 1999 15 699 704 10.1023/A:1008924032039

[B21] Dumestre A Chone T Portal J Gerard M Berthelin J Cyanide degradation under alkaline conditions by a strain of Fusarium solani isolated from contaminated soils Appl Environ Microbiol 1997 63 2729 2734 16535647 10.1128/aem.63.7.2729-2734.1997 PMC1389202

[B22] Bergey DH John GH Bergey’s Manual of Determinative Bacteriology 1994 Baltimore: William and Wilkins

[B23] APHA, AWWA, WPCF Standard Method for the Examination of Water and Wastewater 1995 19 Washington DC: American Public Health Association

[B24] Pyridine-Pyrazalone method for measuring cyanide [http://www.hach.com/asset-get.download.jsa?id=7639983603]

[B25] Das S Santra SC Cyanide degradation by Aspergillus niger strain isolated from steel-plant wastewater Electron J Environ Agri Food Chem 2011 10 7 2516 2522

[B26] Baxter J Cummings SP The impact of bioaugmentation on metal cyanide degradation and soil bacteria community structure Biodegradation 2006 17 207 217 10.1007/s10532-005-4219-6 16715400

[B27] Ezzi MI Lynch JM Biodegradation of cyanide by Trichoderma spp. and Fusarium spp Enzyme Microb Technol 2005 36 7 849 854 10.1016/j.enzmictec.2004.03.030

[B28] Kao CM Liu JK Lou HR Lin CS Chen SC Biotransformation of cyanide to methane and ammonia by Klebsiella oxytoca Chemosphere 2003 50 8 1055 1061 10.1016/S0045-6535(02)00624-0 12531712

[B29] Park D Lee DS Kim YM Park JM Bioaugmentation of cyanide-degrading microorganisms in a full-scale cokes wastewater treatment facility Bioresour Technol 2008 99 2092 2096 10.1016/j.biortech.2007.03.027 17513106

